# Além do Escore CHA2DS2-VASc para Predizer o Risco de Tromboembolismo e Acidente Vascular Cerebral - Não é tão Simples!

**DOI:** 10.36660/abc.20201296

**Published:** 2021-02-19

**Authors:** M. Julia Machline-Carrion

**Affiliations:** ePHealth Primary Care SolutionsSão PauloSPBrasilePHealth Primary Care Solutions, São Paulo, SP - Brasil

**Keywords:** Fatores de Risco, Diabetes, Hipertensão, Aterosclerose, Fibrilação Atrial, Acidente Vascular Cerebral, Embolia e Trombose, Circulação Cerebrovascular

O acidente vascular cerebral (AVC) isquêmico pode ser o resultado de uma variedade de causas, como aterosclerose da circulação cerebral, oclusão de pequenos vasos cerebrais e embolia cardíaca.^1^ Dessas causas, o AVC cardioembólico tem um significado especial, porque a embolia cardíaca causa acidentes vasculares cerebrais mais graves do que outros subtipos de AVC isquêmico.^2^ Em cerca de 20% dos pacientes que tiveram AVC isquêmico, uma fonte principal de risco cardíaco, como fibrilação atrial (FA) e/ou trombos do ventrículo esquerdo, é identificada. Assim, avaliar a presença de FA e o risco de tromboembolismo associado a lesões cardíacas têm um papel fundamental na prevenção do AVC.^3^

Idade, sexo masculino, hipertensão, diabetes mellitus, doença cardíaca valvular, insuficiência cardíaca congestiva, doença cardíaca coronária, doença renal crônica, distúrbios inflamatórios, apneia do sono e uso de tabaco foram estabelecidos como fatores de risco para FA^4^ e acidente vascular cerebral.^5^ No entanto, pode ser que outros fatores atriais além da FA possam resultar em tromboembolismo e, em alguns casos, a FA pode ser um fator retardador marcador dessas outras anormalidades atriais trombogênicas. A FA frequentemente ocorre no contexto de anormalidades atriais, como disfunção mecânica no apêndice atrial esquerdo.^6^ Essas anormalidades do substrato atrial foram recentemente associadas ao risco de AVC independentemente da FA. O escore CHA2DS2-VASc (insuficiência cardíaca congestiva, hipertensão, idade ≥ 75 anos, diabetes mellitus, acidente vascular cerebral ou ataque isquêmico transitório (AIT), doença vascular, idade de 65 a 74 anos, categoria de sexo) é uma ferramenta validada para prever o risco de acidente vascular cerebral e êmbolos sistêmicos em pacientes com fibrilação atrial não-valvular e tem sido amplamente utilizado para orientar a prática clínica.^7-10^

O apêndice atrial esquerdo (AAE) representa uma das principais fontes de trombos cardíacos responsáveis pelo acidente vascular cerebral em pacientes com FA^11^ e isso provavelmente se deve às características anatômicas dessa estrutura, que facilitam o fluxo sanguíneo mais lento em seu interior. Um achado interessante é que a trombose do AAE pode ocorrer mesmo em pacientes com escore CHA2DS2-VASc menor (<2) e isso pode estar relacionado à sua morfologia. A relação entre os achados da ecocardiografia transesofágica e o escore CHA2DS2-VASc ainda não foi estabelecida, uma vez que a maioria dos estudos avalia a associação da presença de trombo com o escore.^12,13^ Linhares et al.,^14^ recentemente apresentaram resultados interessantes sugerindo que a morfologia trombogênica do AAE identificada na ecocardiografia transesofágica (ETE) apresentou maior risco de acidente vascular cerebral, independentemente do escore CHA2DS2-VASc.^14^

Entretanto, não está claro se outro parâmetro, o diâmetro do átrio esquerdo (DAE) combinado com o sistema de escore CHA2DS2-VASc pode melhorar os resultados preditivos de trombose do átrio esquerdo/apêndice atrial esquerdo. O artigo de Zhang Y e Yi Qiang Y,^15^ um estudo retrospectivo incluindo 238 pacientes com fibrilação atrial não valvar, propõe investigar o valor do diâmetro do átrio esquerdo combinado com o escore CHA2DS2-VASc na predição da trombose do átrio esquerdo/apêndice atrial esquerdo na fibrilação atrial não valvar. Os autores descobriram que a análise da curva de característica de operação do receptor revelou que a área sob a curva para o escore CHA2DS2-VASc na previsão de trombose atrial esquerda/trombose do apêndice atrial esquerdo foi 0,593 quando o escore CHA2DS2-VASc era ≥3 pontos, e a sensibilidade e especificidade foram de 86,5% e 32,6%, respectivamente, enquanto a área sob a curva para o DAE na previsão de trombose atrial esquerda/trombose do apêndice atrial esquerdo foi 0,786 quando o DAE era ≥ 44,17 mm, e a sensibilidade e especificidade foram de 89,6% e 60,9%, respectivamente. Além disso, eles descobriram que, entre os diferentes grupos CHA2DS2-VASc, a incidência de trombose atrial esquerda/trombose do apêndice atrial esquerdo em pacientes com DAE ≥ 44,17 mm foi maior do que em pacientes com DAE <44,17 mm.

Em vez de uma resposta definitiva a essa pergunta intrigante, o artigo de Zhang Y e Yi-Qiang Y,^15^ representa principalmente um importante gerador de hipóteses. Os achados são realmente interessantes. No entanto, os leitores devem considerar aspectos importantes que podem limitar a generalização e a aplicação clínica desses achados no momento atual. Primeiro, a sensibilidade do modelo preditivo permaneceu moderada mesmo após a adição do parâmetro DAE; segundo, a especificidade do modelo foi muito baixa para ser sugerida como uma ferramenta padrão; terceiro, devido aos grandes intervalos de confiança observados na análise da associação dos diferentes grupos do escore CHA2DS2-VASc, DAE e ocorrência de trombose, uma considerável incerteza ainda permanece nesses resultados. Finalmente, estudos prospectivos maiores são necessários para entender melhor o papel dos parâmetros cardíacos, como o DAE e o risco de tromboembolismo.

## References

[1] . Adams HP Jr, Bendixen BH, Kappelle LJ, Biller J, Love BB, Gordon DL, et al. Classification of subtype of acute ischemic stroke. Definitions for use in a multicenter clinical trial. TOAST. Trial of Org 10172 in Acute Stroke Treatment. Stroke 1993;24(1):35-41. 10.1161/01.str.24.1.35 7678184

[2] . Lin HJ, Wolf PA, Kelly-Hayes M, Beiser AS, Kase CS, Benjamin EJ, et al. Stroke severity in atrial fibrillation. The Framingham Study. Stroke 1996;27(10):1760-4. 10.1161/01.str.27.10.1760 8841325

[3] . Hart RG, Diener HC, Coutts SB, Easton JD, Granger CB, O’Donnell MJ, et al. Embolic strokes of undetermined source: the case for a new clinical construct. Lancet Neurol.2014;13(4):429-38. 10.1016/S1474-4422(13)70310-7 24646875

[4] . Andrade J, Khairy P, Dobrev D, Nattel S. The clinical profile and pathophysiology of atrial fibrillation: relationships among clinical features, epidemiology, and mechanisms. Circ Res 2014;114(9):1453-68. 10.1161/CIRCRESAHA.114.303211 24763464

[5] . Elkind MS. Inflammatory mechanisms of stroke. Stroke 2010;41(10 Suppl):S3-8. 10.1161/STROKEAHA.110.594945 PMC2963080 20876499

[6] . Warraich HJ, Gandhavadi M, Manning WJ. Mechanical discordance of the left atrium and appendage: a novel mechanism of stroke in paroxysmal atrial fibrillation. Stroke 2014;45(5):1481-4. 10.1161/STROKEAHA.114.004800 24643411

[7] . Lip GY, Nieuwlaat R, Pisters R, Lane DA, Crijns HJ. Refining clinical risk stratification for predicting stroke and thromboembolism in atrial fibrillation using a novel risk factor-based approach: the euro heart survey on atrial fibrillation. Chest 2010;137(2):263-72. 10.1378/chest.09-1584 19762550

[8] . Friberg L, Rosenqvist M, Lip GY. Evaluation of risk stratification schemes for ischaemic stroke and bleeding in 182 678 patients with atrial fibrillation: the Swedish Atrial Fibrillation cohort study. Eur Heart J. 2012;33(12):1500-10. 10.1093/eurheartj/ehr488 22246443

[9] . Mason PK, Lake DE, DiMarco JP, Ferguson JD, Mangrum JM, Bilchick K, et al. Impact of the CHA2DS2-VASc score on anticoagulation recommendations for atrial fibrillation. The Am J Med. 2012;125(6):603 e1-6. 10.1016/j.amjmed.2011.09.030 PMC4555351 22502952

[10] . Olesen JB, Torp-Pedersen C, Hansen ML, Lip GY. The value of the CHA2DS2-VASc score for refining stroke risk stratification in patients with atrial fibrillation with a CHADS2 score 0-1: a nationwide cohort study. Thromb Haemost. 2012;107(6):1172-9. 10.1160/TH12-03-0175 22473219

[11] . Di Biase L, Santangeli P, Anselmino M, Mohanty P, Salvetti I, Gili S, et al. Does the left atrial appendage morphology correlate with the risk of stroke in patients with atrial fibrillation? Results from a multicenter study. J Am Coll Cardiol. 2012;60(6):531-8. 10.1016/j.jacc.2012.04.032 22858289

[12] . Bayard YL, Omran H, Neuzil P, Thuesen L, Pichler M, Rowland E, et al. PLAATO (Percutaneous Left Atrial Appendage Transcatheter Occlusion) for prevention of cardioembolic stroke in non-anticoagulation eligible atrial fibrillation patients: results from the European PLAATO study. EuroIntervention.2010;6(2):220-6. 20562072

[13] . Dawson AG, Asopa S, Dunning J. Should patients undergoing cardiac surgery with atrial fibrillation have left atrial appendage exclusion? Interact Cardiovasc Thorac Surg. 2010;10(2):306-11. 10.1510/icvts.2009.227991 19942634

[14] . LInhares RR, Moreira DAR, Peixoto LB, Cruz AP, Garcia LP, Barreto RB, et al. Association between Morphodynamic Variables by Transesophageal Echocardiography and CHA2DS2-Vasc Values. Int J Cardiovasc Sci. 2019;32(5):460-470.

[15] . Zhang Y e Yi-Qiang Y. Valor do Diâmetro do Átrio Esquerdo com Escore CHA2DS2-Vasc na Predição da Trombose Atrial Esquerda/Trombose de Apêndice Atrial Esquerdo na Fibrilação Atrial Não Valvar. Arq Bras Cardiol. 2021; 116(2):325-331. 10.36660/abc.20190492 PMC7909979 33470330

