# Supplementary material for: Além do Escore CHA2DS2-VASc para Predizer o Risco de Tromboembolismo e Acidente Vascular Cerebral - Não é tão Simples!
Source: Arq Bras Cardiol. 2021 Feb 19;116(2):332–3. [Article in Portuguese] doi: 10.36660/abc.20201296 (PMC7909982; doi:10.36660/abc.20201296)
Supplement: Translation

# Translation: Beyond CHA2DS2-VASc for Predicting the Risk of Thromboembolism and Stroke - Not That Simple!

**Authors:** M. Julia Machline-Carrion

**Affiliations:** BrazilePHealth Primary Care Solutions, São Paulo, SP - Brazil

**Keywords:** Risk Factors, Diabetes, Hypertension, Atherosclerosis, Atrial Fibrillation, Stroke, Embolism and Thrombosis, Cerebrovascular Circulation

Ischemic stroke can result from a variety of causes, such as atherosclerosis of the cerebral circulation, occlusion of cerebral small vessels, and cardiac embolism.^1^ Of these causes, cardioembolic stroke has a particular significance because cardiac embolism causes more severe strokes than other ischemic stroke subtypes.^2^ In about 20% of patients who have had ischemic stroke, a major risk cardiac source, such as atrial fibrillation (AF) and/or left ventricular thrombi, is identified. Thus, assessing the presence of AF, and the risk of thromboembolism associated to cardiac lesions play a key role in stroke prevention. ^3^

Age, male gender, hypertension, diabetes mellitus, valvular heart disease, congestive heart failure, coronary heart disease, chronic kidney disease, inflammatory disorders, sleep apnea, and tobacco use have all been established as risk factors for both AF^4^ and stroke.^5^ However, it may be that other atrial factors besides AF can result in thromboembolism, and in some cases, AF may be a lagging marker of these other thrombogenic atrial abnormalities. AF often occurs in the setting of atrial abnormalities such as mechanical dysfunction in the left atrial appendage.^6^ These abnormalities of atrial substrate have recently been associated with stroke risk independently of AF. The CHA2DS2-VASc (congestive heart failure, hypertension, age ≥ 75 years, diabetes mellitus, stroke or transient ischemic attack (TIA), vascular disease, age 65 to 74 years, sex category) score is a validated tool to predict the risk of stroke and systemic emboli in patients with non-valvular atrial fibrillation and has been widely used to guide clinical practice.^7-10^

Left atrial appendage (LAA) represents one of the main sources of cardiac thrombi responsible for stroke in patients with AF^11^ and this is probably due to the anatomical characteristics of this structure, which facilitate slower blood flow inside it. An interesting finding is that LAA thrombosis can occur even in patients with a lower CHA2DS2VASc score (< 2) and this may be related to its morphology. The relationship between the findings of transesophageal echocardiography and the CHA2DS2-VASc score has not yet been established, since most studies evaluate the association in the presence of thrombus with the score.^12,13^ Linhares et al.,^14^ recently presented interesting results suggesting that the thrombogenic morphology of LAA identified in transesophageal echocardiography (TEE) presented a higher risk of stroke regardless of the CHA2DS2VASc score.^14^

However, it remains unclear whether another parameter, the left atrial diameter (LAD) combined with the CHA2DS2-VASc scoring system can improve the predictive results of left atrial/left atrial appendage thrombosis. Zhang Y e Yi-Qiang Y,^15^ in a retrospective study including 238 patients with non-valvular atrial fibrillation, proposed to investigate the value of left atrial diameter combined with CHA2DS2-VASc score in predicting the left atrial/left atrial appendage thrombosis in non-valvular atrial fibrillation. The authors have found that the receiver operating characteristic curve analysis revealed that the area under the curve for the CHA2DS2-VASc score in predicting left atrial/ left atrial appendage thrombosis was 0.593 when the CHA2DS2-VASc score was ≥ 3 points, and the sensitivity and specificity was 86.5% and 32.6%, respectively, while the area under the curve for LAD in predicting left atrial/ left atrial appendage thrombosis was 0.786 when LAD was ≥ 44.17 mm, and the sensitivity and specificity was 89.6% and 60.9%, respectively. Additionally, they have found that among the different CHA2DS2-VASc groups, the incidence of left atrial/ left atrial appendage thrombosis in patients with a LAD of ≥ 44.17 mm was higher than in patients with a LAD < 44.17 mm.

Rather than a definitive answer to this intriguing question, the paper by Zhang Y and Yi-Qiang Y^15^ mostly represents an important hypothesis generator. The findings are interesting indeed. However, the readers should consider important aspects that may limit the generalizability and clinical application of these findings at the present moment. First, the sensitivity of the predictive model remained moderate even after the addition of the LAD parameter; second, the specificity of the model was too low to be suggested as a standard tool; third, due to the large confidence intervals observed in the analysis of the association of the different different CHA2DS2-VASc groups, LAD and occurrence of thrombosis, a considerable uncertainty still remains in these results. Finally, larger prospective studies are needed to better understand the role of cardiac parameters, such as LAD and the risk of thromboembolism.
